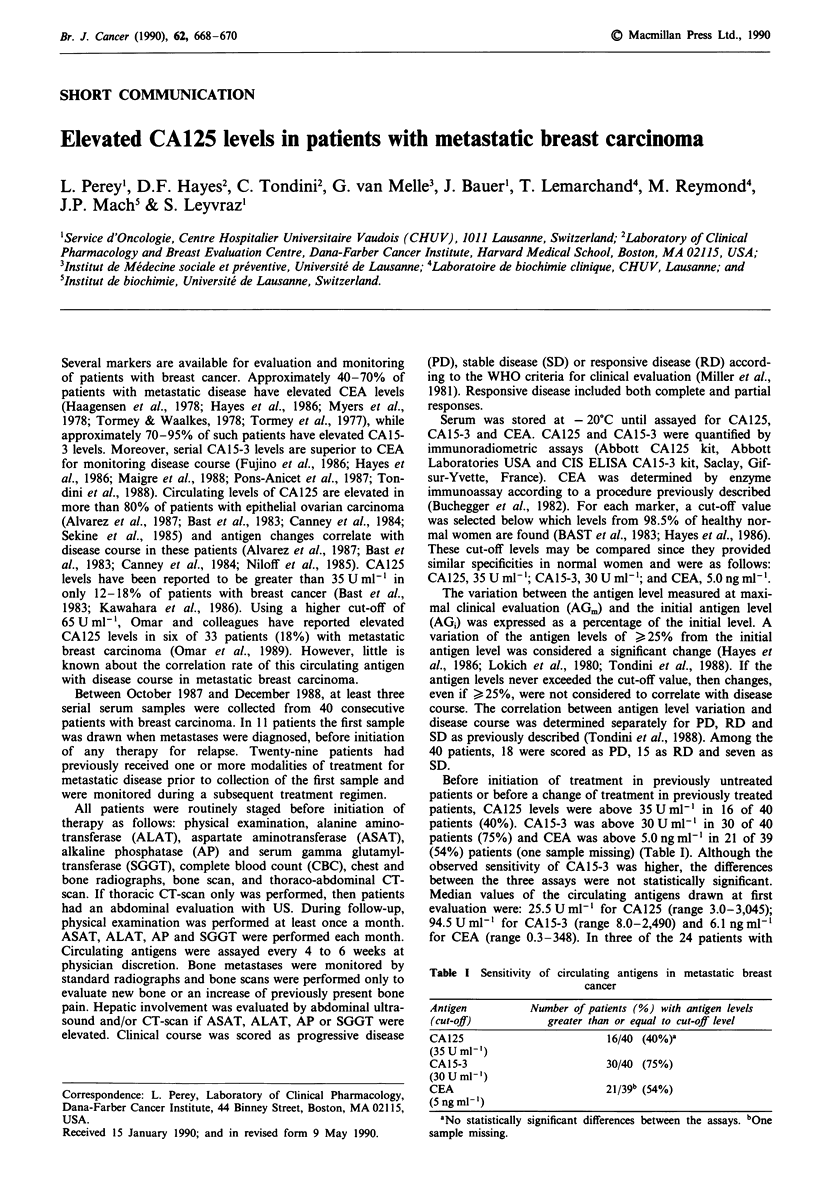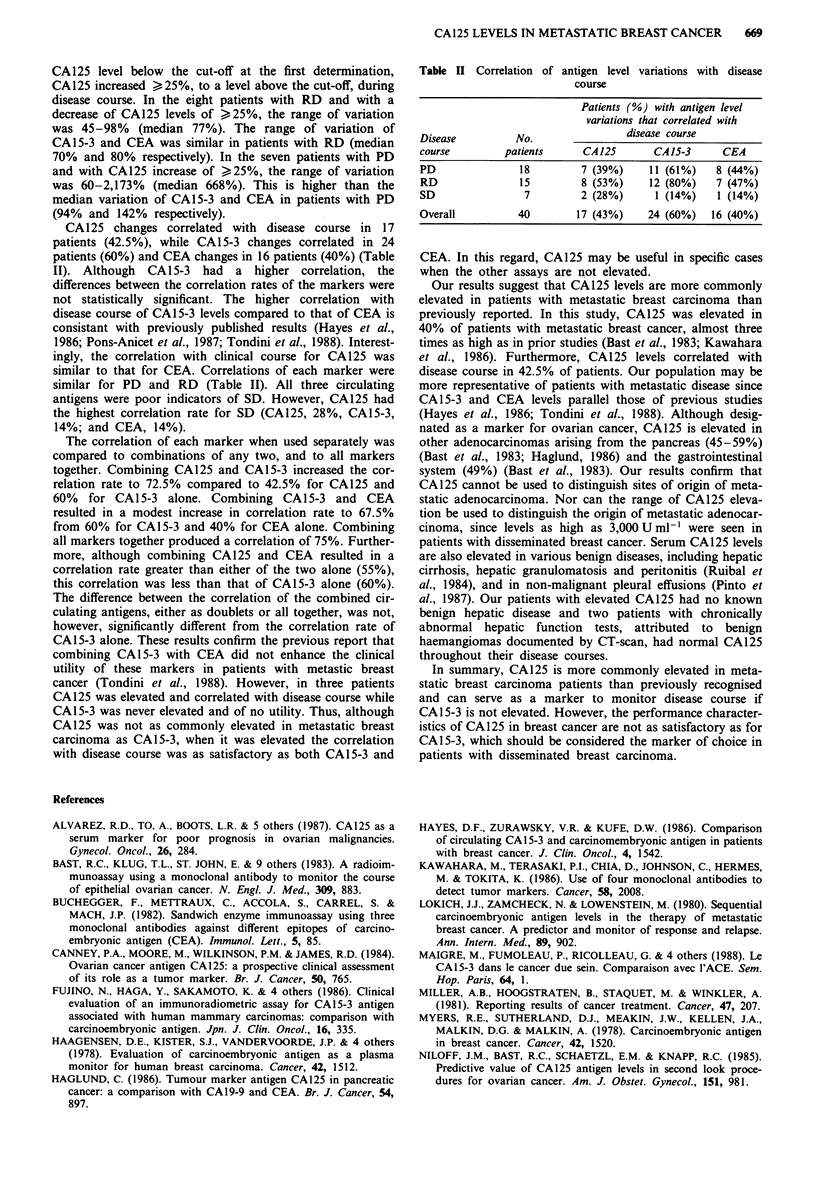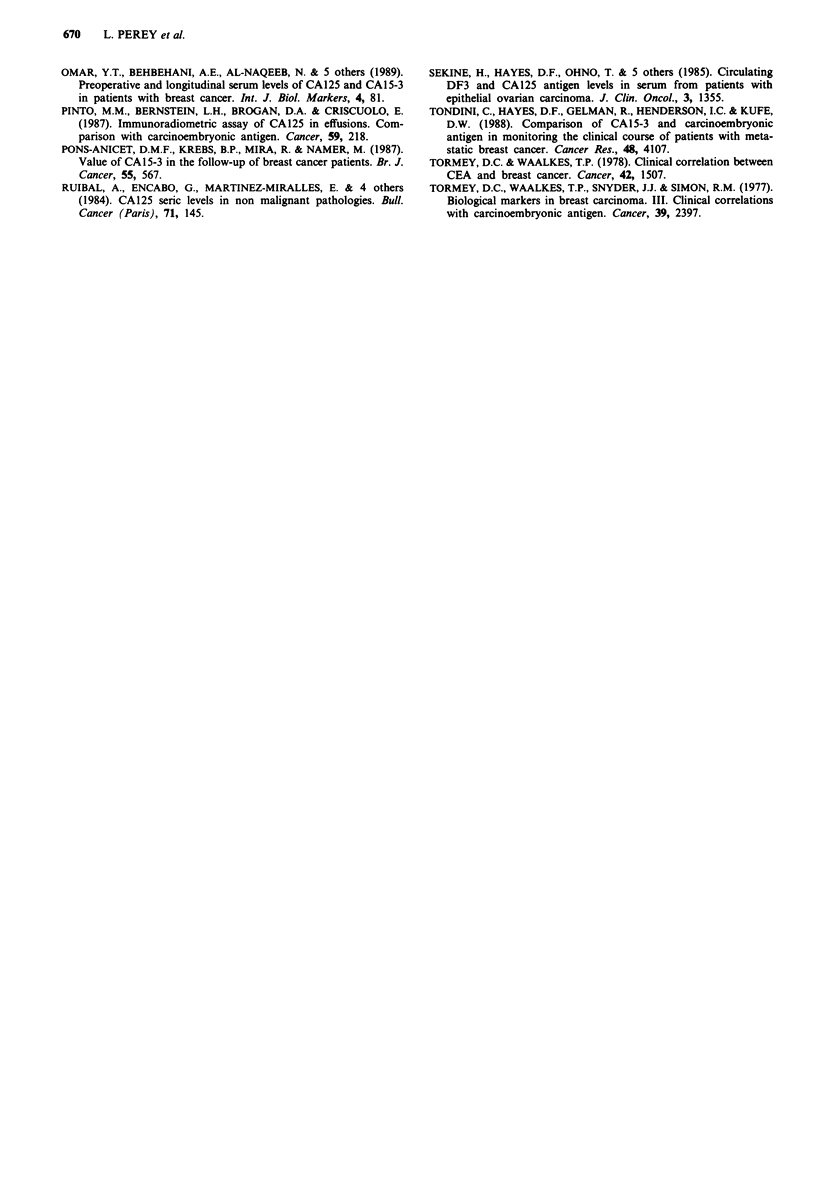# Elevated CA125 levels in patients with metastatic breast carcinoma.

**DOI:** 10.1038/bjc.1990.353

**Published:** 1990-10

**Authors:** L. Perey, D. F. Hayes, C. Tondini, G. van Melle, J. Bauer, T. Lemarchand, M. Reymond, J. P. Mach, S. Leyvraz

**Affiliations:** Service d'Oncologie, Centre Hospitalier Universitaire Vaudois (CHUV), Lausanne, Switzerland.


Br. J. Cancer (1990), 62, 668-670 © Macmillan Press Ltd., 1990

SHORT COMMUNICATION

### Elevated CA125 levels in patients with metastatic breast carcinoma

L. Perey[1], D.F. Hayes[2], C. Tondini[2], G. van Melle[3], J. Bauer[1], T. Lemarchand[4], M. Reymond[4], J.P. Mach[5] & S. Leyvraz[1]

[1]*Service d'Oncologie, Centre Hospitalier Universitaire Vaudois (CHUV), 1011 Lausanne, Switzerland;* [2]*Laboratory of Clinical Pharmacology and Breast Evaluation Centre, Dana-Farber Cancer Institute, Harvard Medical School, Boston, MA 02115, USA;* [3]*Institut de Médecine sociale et préventive, Université de Lausanne;* [4]*Laboratoire de biochimie clinique, CHUV, Lausanne; and* [5]*Institut de biochimie, Université de Lausanne, Switzerland.*

Several markers are available for evaluation and monitoring of patients with breast cancer. Approximately 40–70% of patients with metastatic disease have elevated CEA levels (Haagensen *et al.*, 1978; Hayes *et al.*, 1986; Myers *et al.*, 1978; Tormey & Waalkes, 1978; Tormey *et al.*, 1977), while approximately 70–95% of such patients have elevated CA15-3 levels. Moreover, serial CA15-3 levels are superior to CEA for monitoring disease course (Fujino *et al.*, 1986; Hayes *et al.*, 1986; Maigre *et al.*, 1988; Pons-Anicet *et al.*, 1987; Tondini *et al.*, 1988). Circulating levels of CA125 are elevated in more than 80% of patients with epithelial ovarian carcinoma (Alvarez *et al.*, 1987; Bast *et al.*, 1983; Canney *et al.*, 1984; Sekine *et al.*, 1985) and antigen changes correlate with disease course in these patients (Alvarez *et al.*, 1987; Bast *et al.*, 1983; Canney *et al.*, 1984; Niloff *et al.*, 1985). CA125 levels have been reported to be greater than 35 U $ml^{-1}$ in only 12–18% of patients with breast cancer (Bast *et al.*, 1983; Kawahara *et al.*, 1986). Using a higher cut-off of 65 U $ml^{-1}$, Omar and colleagues have reported elevated CA125 levels in six of 33 patients (18%) with metastatic breast carcinoma (Omar *et al.*, 1989). However, little is known about the correlation rate of this circulating antigen with disease course in metastatic breast carcinoma.

Between October 1987 and December 1988, at least three serial serum samples were collected from 40 consecutive patients with breast carcinoma. In 11 patients the first sample was drawn when metastases were diagnosed, before initiation of any therapy for relapse. Twenty-nine patients had previously received one or more modalities of treatment for metastatic disease prior to collection of the first sample and were monitored during a subsequent treatment regimen.

All patients were routinely staged before initiation of therapy as follows: physical examination, alanine aminotransferase (ALAT), aspartate aminotransferase (ASAT), alkaline phosphatase (AP) and serum gamma glutamyltransferase (SGGT), complete blood count (CBC), chest and bone radiographs, bone scan, and thoraco-abdominal CT-scan. If thoracic CT-scan only was performed, then patients had an abdominal evaluation with US. During follow-up, physical examination was performed at least once a month. ASAT, ALAT, AP and SGGT were performed each month. Circulating antigens were assayed every 4 to 6 weeks at physician discretion. Bone metastases were monitored by standard radiographs and bone scans were performed only to evaluate new bone or an increase of previously present bone pain. Hepatic involvement was evaluated by abdominal ultrasound and/or CT-scan if ASAT, ALAT, AP or SGGT were elevated. Clinical course was scored as progressive disease (PD), stable disease (SD) or responsive disease (RD) according to the WHO criteria for clinical evaluation (Miller *et al.*, 1981). Responsive disease included both complete and partial responses.

Serum was stored at − 20°C until assayed for CA125, CA15-3 and CEA. CA125 and CA15-3 were quantified by immunoradiometric assays (Abbott CA125 kit, Abbott Laboratories USA and CIS ELISA CA15-3 kit, Saclay, Gif-sur-Yvette, France). CEA was determined by enzyme immunoassay according to a procedure previously described (Buchegger *et al.*, 1982). For each marker, a cut-off value was selected below which levels from 98.5% of healthy normal women are found (BAST *et al.*, 1983; Hayes *et al.*, 1986). These cut-off levels may be compared since they provided similar specificities in normal women and were as follows: CA125, 35 U $ml^{-1}$; CA15-3, 30 U $ml^{-1}$; and CEA, 5.0 ng $ml^{-1}$.

The variation between the antigen level measured at maximal clinical evaluation ($AG_m$) and the initial antigen level ($AG_i$) was expressed as a percentage of the initial level. A variation of the antigen levels of $\geqslant$25% from the initial antigen level was considered a significant change (Hayes *et al.*, 1986; Lokich *et al.*, 1980; Tondini *et al.*, 1988). If the antigen levels never exceeded the cut-off value, then changes, even if $\geqslant$25%, were not considered to correlate with disease course. The correlation between antigen level variation and disease course was determined separately for PD, RD and SD as previously described (Tondini *et al.*, 1988). Among the 40 patients, 18 were scored as PD, 15 as RD and seven as SD.

Before initiation of treatment in previously untreated patients or before a change of treatment in previously treated patients, CA125 levels were above 35 U $ml^{-1}$ in 16 of 40 patients (40%). CA15-3 was above 30 U $ml^{-1}$ in 30 of 40 patients (75%) and CEA was above 5.0 ng $ml^{-1}$ in 21 of 39 (54%) patients (one sample missing) (Table I). Although the observed sensitivity of CA15-3 was higher, the differences between the three assays were not statistically significant. Median values of the circulating antigens drawn at first evaluation were: 25.5 U $ml^{-1}$ for CA125 (range 3.0–3,045); 94.5 U $ml^{-1}$ for CA15-3 (range 8.0–2,490) and 6.1 ng $ml^{-1}$ for CEA (range 0.3–348). In three of the 24 patients with

**Table I** Sensitivity of circulating antigens in metastatic breast cancer

| *Antigen (cut-off)* | *Number of patients (%) with antigen levels greater than or equal to cut-off level* |
|---|---|
| CA125 (35 U $ml^{-1}$) | 16/40 (40%)[a] |
| CA15-3 (30 U $ml^{-1}$) | 30/40 (75%) |
| CEA (5 ng $ml^{-1}$) | 21/39[b] (54%) |

[a]No statistically significant differences between the assays. [b]One sample missing.

Correspondence: L. Perey, Laboratory of Clinical Pharmacology, Dana-Farber Cancer Institute, 44 Binney Street, Boston, MA 02115, USA.
Received 15 January 1990; and in revised form 9 May 1990.

CA125 level below the cut-off at the first determination, CA125 increased ⩾25%, to a level above the cut-off, during disease course. In the eight patients with RD and with a decrease of CA125 levels of ⩾25%, the range of variation was 45–98% (median 77%). The range of variation of CA15-3 and CEA was similar in patients with RD (median 70% and 80% respectively). In the seven patients with PD and with CA125 increase of ⩾25%, the range of variation was 60–2,173% (median 668%). This is higher than the median variation of CA15-3 and CEA in patients with PD (94% and 142% respectively).

CA125 changes correlated with disease course in 17 patients (42.5%), while CA15-3 changes correlated in 24 patients (60%) and CEA changes in 16 patients (40%) (Table II). Although CA15-3 had a higher correlation, the differences between the correlation rates of the markers were not statistically significant. The higher correlation with disease course of CA15-3 levels compared to that of CEA is consistant with previously published results (Hayes *et al.*, 1986; Pons-Anicet *et al.*, 1987; Tondini *et al.*, 1988). Interestingly, the correlation with clinical course for CA125 was similar to that for CEA. Correlations of each marker were similar for PD and RD (Table II). All three circulating antigens were poor indicators of SD. However, CA125 had the highest correlation rate for SD (CA125, 28%, CA15-3, 14%; and CEA, 14%).

The correlation of each marker when used separately was compared to combinations of any two, and to all markers together. Combining CA125 and CA15-3 increased the correlation rate to 72.5% compared to 42.5% for CA125 and 60% for CA15-3 alone. Combining CA15-3 and CEA resulted in a modest increase in correlation rate to 67.5% from 60% for CA15-3 and 40% for CEA alone. Combining all markers together produced a correlation of 75%. Furthermore, although combining CA125 and CEA resulted in a correlation rate greater than either of the two alone (55%), this correlation was less than that of CA15-3 alone (60%). The difference between the correlation of the combined circulating antigens, either as doublets or all together, was not, however, significantly different from the correlation rate of CA15-3 alone. These results confirm the previous report that combining CA15-3 with CEA did not enhance the clinical utility of these markers in patients with metastic breast cancer (Tondini *et al.*, 1988). However, in three patients CA125 was elevated and correlated with disease course while CA15-3 was never elevated and of no utility. Thus, although CA125 was not as commonly elevated in metastatic breast carcinoma as CA15-3, when it was elevated the correlation with disease course was as satisfactory as both CA15-3 and CEA. In this regard, CA125 may be useful in specific cases when the other assays are not elevated.

**Table II** Correlation of antigen level variations with disease course

| Disease course | No. patients | *Patients (%) with antigen level variations that correlated with disease course* CA125 | CA15-3 | CEA |
|---|---|---|---|---|
| PD | 18 | 7 (39%) | 11 (61%) | 8 (44%) |
| RD | 15 | 8 (53%) | 12 (80%) | 7 (47%) |
| SD | 7 | 2 (28%) | 1 (14%) | 1 (14%) |
| Overall | 40 | 17 (43%) | 24 (60%) | 16 (40%) |

Our results suggest that CA125 levels are more commonly elevated in patients with metastatic breast carcinoma than previously reported. In this study, CA125 was elevated in 40% of patients with metastatic breast cancer, almost three times as high as in prior studies (Bast *et al.*, 1983; Kawahara *et al.*, 1986). Furthermore, CA125 levels correlated with disease course in 42.5% of patients. Our population may be more representative of patients with metastatic disease since CA15-3 and CEA levels parallel those of previous studies (Hayes *et al.*, 1986; Tondini *et al.*, 1988). Although designated as a marker for ovarian cancer, CA125 is elevated in other adenocarcinomas arising from the pancreas (45–59%) (Bast *et al.*, 1983; Haglund, 1986) and the gastrointestinal system (49%) (Bast *et al.*, 1983). Our results confirm that CA125 cannot be used to distinguish sites of origin of metastatic adenocarcinoma. Nor can the range of CA125 elevation be used to distinguish the origin of metastatic adenocarcinoma, since levels as high as 3,000 U $ml^{-1}$ were seen in patients with disseminated breast cancer. Serum CA125 levels are also elevated in various benign diseases, including hepatic cirrhosis, hepatic granulomatosis and peritonitis (Ruibal *et al.*, 1984), and in non-malignant pleural effusions (Pinto *et al.*, 1987). Our patients with elevated CA125 had no known benign hepatic disease and two patients with chronically abnormal hepatic function tests, attributed to benign haemangiomas documented by CT-scan, had normal CA125 throughout their disease courses.

In summary, CA125 is more commonly elevated in metastatic breast carcinoma patients than previously recognised and can serve as a marker to monitor disease course if CA15-3 is not elevated. However, the performance characteristics of CA125 in breast cancer are not as satisfactory as for CA15-3, which should be considered the marker of choice in patients with disseminated breast carcinoma.

### References

ALVAREZ, R.D., TO, A., BOOTS, L.R. & 5 others (1987). CA125 as a serum marker for poor prognosis in ovarian malignancies. *Gynecol. Oncol.*, **26**, 284.

BAST, R.C., KLUG, T.L., ST. JOHN, E. & 9 others (1983). A radioimmunoassay using a monoclonal antibody to monitor the course of epithelial ovarian cancer. *N. Engl. J. Med.*, **309**, 883.

BUCHEGGER, F., METTRAUX, C., ACCOLA, S., CARREL, S. & MACH, J.P. (1982). Sandwich enzyme immunoassay using three monoclonal antibodies against different epitopes of carcinoembryonic antigen (CEA). *Immunol. Lett.*, **5**, 85.

CANNEY, P.A., MOORE, M., WILKINSON, P.M. & JAMES, R.D. (1984). Ovarian cancer antigen CA125: a prospective clinical assessment of its role as a tumor marker. *Br. J. Cancer*, **50**, 765.

FUJINO, N., HAGA, Y., SAKAMOTO, K. & 4 others (1986). Clinical evaluation of an immunoradiometric assay for CA15-3 antigen associated with human mammary carcinomas: comparison with carcinoembryonic antigen. *Jpn. J. Clin. Oncol.*, **16**, 335.

HAAGENSEN, D.E., KISTER, S.J., VANDERVOORDE, J.P. & 4 others (1978). Evaluation of carcinoembryonic antigen as a plasma monitor for human breast carcinoma. *Cancer*, **42**, 1512.

HAGLUND, C. (1986). Tumour marker antigen CA125 in pancreatic cancer: a comparison with CA19-9 and CEA. *Br. J. Cancer*, **54**, 897.

HAYES, D.F., ZURAWSKY, V.R. & KUFE, D.W. (1986). Comparison of circulating CA15-3 and carcinomembryonic antigen in patients with breast cancer. *J. Clin. Oncol.*, **4**, 1542.

KAWAHARA, M., TERASAKI, P.I., CHIA, D., JOHNSON, C., HERMES, M. & TOKITA, K. (1986). Use of four monoclonal antibodies to detect tumor markers. *Cancer*, **58**, 2008.

LOKICH, J.J., ZAMCHECK, N. & LOWENSTEIN, M. (1980). Sequential carcinoembryonic antigen levels in the therapy of metastatic breast cancer. A predictor and monitor of response and relapse. *Ann. Intern. Med.*, **89**, 902.

MAIGRE, M., FUMOLEAU, P., RICOLLEAU, G. & 4 others (1988). Le CA15-3 dans le cancer due sein. Comparaison avec l'ACE. *Sem. Hop. Paris*, **64**, 1.

MILLER, A.B., HOOGSTRATEN, B., STAQUET, M. & WINKLER, A. (1981). Reporting results of cancer treatment. *Cancer*, **47**, 207.

MYERS, R.E., SUTHERLAND, D.J., MEAKIN, J.W., KELLEN, J.A., MALKIN, D.G. & MALKIN, A. (1978). Carcinoembryonic antigen in breast cancer. *Cancer*, **42**, 1520.

NILOFF, J.M., BAST, R.C., SCHAETZL, E.M. & KNAPP, R.C. (1985). Predictive value of CA125 antigen levels in second look procedures for ovarian cancer. *Am. J. Obstet. Gynecol.*, **151**, 981.

OMAR, Y.T., BEHBEHANI, A.E., AL-NAQEEB, N. & 5 others (1989). Preoperative and longitudinal serum levels of CA125 and CA15-3 in patients with breast cancer. *Int. J. Biol. Markers*, **4**, 81.

PINTO, M.M., BERNSTEIN, L.H., BROGAN, D.A. & CRISCUOLO, E. (1987). Immunoradiometric assay of CA125 in effusions. Comparison with carcinoembryonic antigen. *Cancer*, **59**, 218.

PONS-ANICET, D.M.F., KREBS, B.P., MIRA, R. & NAMER, M. (1987). Value of CA15-3 in the follow-up of breast cancer patients. *Br. J. Cancer*, **55**, 567.

RUIBAL, A., ENCABO, G., MARTINEZ-MIRALLES, E. & 4 others (1984). CA125 seric levels in non malignant pathologies. *Bull. Cancer (Paris)*, **71**, 145.

SEKINE, H., HAYES, D.F., OHNO, T. & 5 others (1985). Circulating DF3 and CA125 antigen levels in serum from patients with epithelial ovarian carcinoma. *J. Clin. Oncol.*, **3**, 1355.

TONDINI, C., HAYES, D.F., GELMAN, R., HENDERSON, I.C. & KUFE, D.W. (1988). Comparison of CA15-3 and carcinoembryonic antigen in monitoring the clinical course of patients with metastatic breast cancer. *Cancer Res.*, **48**, 4107.

TORMEY, D.C. & WAALKES, T.P. (1978). Clinical correlation between CEA and breast cancer. *Cancer*, **42**, 1507.

TORMEY, D.C., WAALKES, T.P., SNYDER, J.J. & SIMON, R.M. (1977). Biological markers in breast carcinoma. III. Clinical correlations with carcinoembryonic antigen. *Cancer*, **39**, 2397.